# Frequency Distributions of Alleles and Genotypes and Lung Cancer Risk of Polymorphisms DCK, SLC29A1, and SLC29A3 in South Indian Healthy Population

**DOI:** 10.7759/cureus.71896

**Published:** 2024-10-19

**Authors:** Devika T, Ganesapandian Mahalakshmi, K Mythili, Katiboina Srinivasa Rao, Srinivasamurthy Suresh Kumar, Biswajit Dubashi, Deepak G Shewade

**Affiliations:** 1 Department of Pharamcology, Guntur Medical College, Guntur, IND; 2 Department of Pharmacology, Nandha Medical College and Hospital, Erode, IND; 3 Department of Physiology, Government Siddhartha Medical College, Vijayawada, IND; 4 Department of Pharmacology, All India Institute of Medical Sciences, Mangalagiri, IND; 5 Department of Pharmacology, Ras Al Khaimah College of Medical Sciences, Ras Al Khaimah Medical and Health Sciences University, Ras Al Khaimah, ARE; 6 Department of Medical Oncology, Jawaharlal Institute of Postgraduate Medical Education and Research, Puducherry, IND; 7 Department of Pharmacology, Jawaharlal Institute of Postgraduate Medical Education and Research, Puducherry, IND

**Keywords:** dck, gemcitabine, polymorphisms, slc29a1, slc29a3, snps

## Abstract

Introduction

Gemcitabine, a cytotoxic drug, is used to treat a variety of solid tumors, such as pancreatic, lung, and breast malignancies. The efficiency rates for gemcitabine have decreased due to an increase in genetic instability. The association between gene polymorphisms and the efficacy of gemcitabine therapy may be better known by understanding the intricacies of genetics that target a few or more genes in drug-targeting metabolic pathways. Moreover, several studies have documented differences in the therapeutic response among various ethnicities to gemcitabine chemotherapy. Therefore, the purpose of this study was to determine the normative frequencies of gene polymorphisms linked to the metabolic pathway of gemcitabine (*DCK* -360C>G (80143932), *SLC29A1* -201A>G (760370), *SLC29A1* +913C>T (9394992), *SLC29A3* +4967C>A (10999776)) in Southern part of Indian healthy population and compared it with the 1000 genome population. In addition, the association of the above single nucleotide polymorphisms (SNPs) with lung cancer susceptibility was also evaluated.

Methods

The present study used real-time polymerase chain reaction (RT-PCR) for performing genotyping in 184 healthy participants as well as 123 South Indian patients with lung cancer. The frequencies of alleles and genotypes of the aforementioned genetic variants were in Hardy-Weinberg equilibrium (p > 0.05).

Results

The minor allele frequencies (MAF) of the SNPs *DCK* -360C>G (80143932), *SLC29A1* -201A>G (760370), *SLC29A1*+913C>T (9394992), *SLC29A3* +4967C>A (10999776) were 3.8%, 17.7%, 27.7%, 29.3% respectively in healthy population. The MAF of the SNPs, *DCK* -360C>G (80143932), *SLC29A1* -201A>G (760370), *SLC29A1* +913C>T (9394992), *SLC29A3* +4967C>A (10999776) in lung cancer patients was 2%, 15%, 23.2%, and 24.4% respectively. A trend toward a protective effect against lung cancer was observed with *SLC29A1 *+913C>T (9394992).

Conclusion

The observed frequencies of alleles and genotypes in the South Indian population were significantly different as compared to the 1000 genome population. In the present study, an association of SLC29A1 rs9394992 C>T between lung cancer patients and healthy subjects showed a trend toward protective effect against lung cancer risk. There was no association found between the other studied SNPs and lung cancer risk.

## Introduction

There are about 3.2 billion base pairs in the human genome, of which 99.9% of DNA sequence is similar among individuals across the population; the remaining accounts for genetic polymorphisms. Frequent or repeated mutations occurring at a particular locus are known as a genetic polymorphism. Pharmacogenetic studies evince that genetic variants that are involved in gemcitabine pharmacology may serve as useful markers for predicting responses to treatment across interpatient and interethnic groups.

Deoxycytidine kinase (*DCK*) is believed to be one of the major factors in assessing the sensitivity to gemcitabine. It is an important enzyme that recycles the preformed nucleosides in the synthesis of DNA. *DCK* phosphorylates deoxyribonucleosides to their respective deoxyribonucleotides by specifically adding the first phosphoryl group. Having structural similarity with nucleosides, *DCK* phosphorylates the exogenously administered gemcitabine by monophosphorylation, which is a crucial process in the activation of gemcitabine. Tumor tissues, when exposed to prolonged concentrations of gemcitabine, were found to be associated with reduced *DCK* expression, and it was also associated with the development of acquired resistance to gemcitabine [1,2]. Though there is a lack of evidence to show an association between *DCK* single nucleotide polymorphisms (SNPs) and positive outcomes in patients treated with gemcitabine, there exists a well-established correlation in patients treated with cytosine arabinoside (Ara-C) [3]. This anti-cancer drug also gets metabolized in a similar fashion as gemcitabine. It was shown that the haplotype GT, including *DCK* rs80143932 and rs2306744, was associated with complete remission and six months of relapse-free survival [4]. Ethnically, this haplotype had a higher frequency in patients of Asian origin and lower in patients of Caucasian origin (15.6% and 2%, respectively); further, it is also found to be associated with elevated expression of *DCK* levels both in vitro as well as in vivo [4,5]. Ethnic differences among different populations in *DCK* -360C>G (80143932) were reported [6].

Gemcitabine is transported intracellularly by various drug transporters. One of the main transporters includes the solute carrier transporter. Solute carrier family 29 member 1 (*SLC29A1*) encodes a transmembrane protein that spans through the plasma membrane and allows the transport of drugs through facilitated diffusion. They are also involved in nucleotide synthesis in the cells that lack the de novo pathway. Multiple alternatively spliced variants are found for this gene. Cellular uptake of gemcitabine by *SLC29A1* has been confirmed in in vitro studies, and the cells lacking this transporter are highly resistant to gemcitabine [7]. Being a major transporter involved in gemcitabine transport, it helps in predicting the response rates in patients who received gemcitabine-based regimens for various types of cancer. Many studies provide much evidence of increased levels of *SLC29A1* and improved survival outcomes in individuals on gemcitabine therapy. Two meta-analyses, including 875 pancreatic patients from 12 studies and 632 patients from 16 studies, respectively, have reported that elevated expression levels of gene *SLC29A1* were associated with improved outcomes [8-11]. Another important transporter, solute carrier family 29 member 3 (*SLC29A3*), has also been found to play an important role in patient outcomes when treated with gemcitabine [12,13]. The SNP rs10999776 C>T was studied to find out the association with survival outcomes in non-small-cell lung cancer (NSCLC) patients on gemcitabine. The genotypes CT and TT had longer survival as compared to the CC genotype [14].

Evidence from the literature indicates that variations in drug-metabolizing pathways have impeded desired clinical results. Resistance to drug therapy can be addressed by understanding the variability in genetic makeup and help in diagnosis and planning treatment protocols [15,16]. Various factors have been implicated in determining the response as well as the risk of gemcitabine toxicity [17,18]. Of these, SNPs in the metabolic pathway of gemcitabine take a crucial role in determining the prognosis, treatment outcome, and drug-induced adverse reactions. SNPs of *DCK* and *SLC* transporters have been implicated in the inter-individual variability in the treatment outcomes of gemcitabine-based therapy in terms of therapeutic response and toxicity [19]. In this context, the detection of such genetic polymorphic variants can serve as a pharmacogenetic marker in patients eligible for gemcitabine-based therapy [20,21]. Additionally, the frequencies of polymorphic alleles in these genes vary considerably among global populations [22]. Based on this backdrop, we undertook a pharmacogenetic association analysis with genetic variations in the metabolic pathway genes in south Indian healthy and lung cancer patients on gemcitabine-based chemotherapy.

## Materials and methods

This study was done in the Department of Pharmacology, Jawaharlal Institute of Postgraduate Medical Education and Research (JIPMER), Puducherry, along with the Department of Medical Oncology, Regional Cancer Centre (RCC), JIPMER. Inclusion criteria for healthy subjects were participants (N = 184) aged between 18 and 65 years of either gender, with no history of cancer, and also belonging to South Indian origin. Inclusion criteria for lung cancer patients are stage IV metastatic lung cancer patients (N = 123) who were prescribed gemcitabine-based chemotherapy aged between 18 and 65 years; both sexes were recruited. Exclusion criteria for both healthy volunteers and lung cancer patients were subjects with any liver or kidney dysfunction; pregnant and lactating women were excluded from the study. The study was started after getting approval from the Institute Ethics Committee (JIP/IEC/2014/4/310). Informed consent was taken from all the study participants. DNA extraction was done for both healthy and lung cancer patient samples using leukocyte fraction of the blood by phenol-chloroform extraction procedures, and genotyping was done using RT-PCR techniques to identify gene polymorphisms. The genotype and allele data of healthy subjects were used for frequency establishment and to compare with 1000 genome population, International Genome Sample Resource (IGSR) (https://www.internationalgenome.org/). Additionally, the genotype and allele frequency data from lung cancer patients was used to find cancer risk between healthy and lung cancer patients. Statistical analysis was done using SPSS version 19.0 (IBM SPSS Statistics for Windows, IBM Corp., Armonk, NY) and GraphPad InStat version 3.06 (GraphPad Software, San Diego, CA). The two-sided p-value of < 0.05 was considered significant. Frequencies were analyzed using the direct gene count method. For Hardy-Weinberg equilibrium and frequency comparison (using data from the 1000 genome project, the chi-square (χ^2^) test was used. For analyzing the linkage disequilibrium, the penalized-likelihood expectation maximization (PLEM) algorithm with HaploView software version 4.2 (Dr. Mark Daly, MIT/Harvard Broad Institute, Cambridge, MA) was used. A case-control analysis was done using binary logistic regression.

## Results

Genotype and allele frequencies in healthy population

DCK C>G (rs80143932)

The genotype frequencies are CC 93.5%, CG 10%, and GG 2%, while the allele frequencies are C 96.2% and G 3.8%. A significant difference was observed in the frequencies of *DCK* and of AFR (African), EAS (East Asians), EUR (Europeans), and BEB (Bengali in Bangladesh) and are similar to AMR (American) and SAS (South Asians) populations (Table 1).

**Table 1:** Comparison of the genotype and allele frequencies of DCK rs80143932 C>G polymorphism in healthy population with 1000 genome populations *p < 0.05 was considered to be significant. AFR, African; AMR, American; BEB, Bengali in Bangladesh; EAS, East Asians; EUR, Europeans; GIH, Gujarati Indians in Houston, TX; ITU, Indian Telugu in the UK; N, number of subjects; PJL, Punjabi in Lahore; SAS, South Asians; SI, South Indian; STU, Sri Lankan Tamils in the UK

Present	N	Genotype frequency (%)			Allele frequency(%)		p-value
		CC	CG	GG	C	G	
SI	184	93.5	5.4	1.1	96.2	3.8	Ref
AFR	661	97.0	2.9	0.1	98.4	1.6	0.008*
AMR	347	90.2	9.2	0.6	94.8	5.2	0.3113
EAS	504	71.6	25.2	3.2	84.2	15.8	<0.0001*
EUR	503	97.8	2.2	0	98.9	1.1	0.0009*
SAS							
BEB	86	81.4	18.6	0	90.7	9.3	0.009*
GIH	103	89.3	10.7	0	94.7	5.3	0.387
ITU	102	93.1	6.9	0	96.6	3.4	0.820
PJL	96	92.7	6.2	0.1	95.8	4.2	0.834
STU	102	91.2	7.8	0.1	95.1	4.9	0.530

SLC29A1 A>G (rs760370)

The major allele A was found to be 82.3%, whereas G is 17.7%. The homozygous wild type, i.e., AA was 67.9%, heterozygous AG was 28.8%, and homozygous mutant was 6%. The frequencies were different from the populations of 1000 genome projects, including AFR, AMR, EAS, EUR, and GIH (Table 2).

**Table 2:** Comparison of the genotype and allele frequencies of SLC29A1 rs760370 A>G polymorphism in healthy population with 1000 genome populations *p < 0.05 was considered to be significant. AFR, African; AMR, American; BEB, Bengali in Bangladesh; EAS, East Asians; EUR, Europeans; GIH, Gujarati Indians in Houston, TX; ITU, Indian Telugu in the UK; N, number of subjects; PJL, Punjabi in Lahore; SAS, South Asians; SI, South Indian; STU, Sri Lankan Tamils in the UK

Present	N	Genotype frequency (%)			Allele frequency(%)		p-value
		AA	AG	GG	A	G	
SI	184	67.9	28.8	3.3	82.3	17.7	Ref
AFR	661	54.6	38.3	7.1	73.8	26.2	0.0007*
AMR	347	40.6	43.8	15.6	62.5	37.5	<0.0001*
EAS	504	48.8	42.7	8.5	70.1	29.9	<0.0001*
EUR	503	37.8	44.9	17.3	60.2	39.8	<0.0001*
SAS							
BEB	86	66.3	33.7	0	83.1	16.9	0.818
GIH	103	56.3	35.0	8.7	73.8	26.2	0.015*
ITU	102	67.6	28.4	3.9	81.9	18.1	0.887
PJL	96	65.6	29.2	5.2	80.2	19.8	0.537
STU	102	70.6	24.5	4.9	82.8	17.2	0.878

SLC29A1 C>T (rs9394992)

The frequency of C (72.3%) and the frequency of T (27.7%) were significantly divergent from EAS and PJL (Punjabi in Lahore) of the South Asian subpopulation. The genotype frequencies for CC, CT, and TT were 51.6%, 41.3%, and 7.1%, respectively (Table 3).

**Table 3:** Comparison of the genotype and allele frequencies of SLC29A1 rs9394992 C>T polymorphism in healthy population with 1000 genome populations *p < 0.05 was considered to be significant. AFR, African; AMR, American; BEB, Bengali in Bangladesh; EAS, East Asians; EUR, Europeans; GIH, Gujarati Indians in Houston, TX; ITU, Indian Telugu in the UK; N, number of subjects; PJL, Punjabi in Lahore; SAS, South Asians; SI, South Indian; STU, Sri Lankan Tamils in the UK

Present	N	Genotype frequency (%)			Allele frequency(%)		p-value
		CC	CT	TT	C	T	
SI	184	51.6	41.3	7.1	72.3	27.7	Ref
AFR	661	46.1	45.1	8.8	68.7	31.3	0.184
AMR	347	57.1	36.9	6.1	75.5	24.5	0.252
EAS	504	40.1	46.0	13.9	63.1	36.9	0.001*
EUR	503	51.7	39.8	8.5	71.6	28.4	0.795
SAS							
BEB	86	57.0	36.0	7.0	75.0	25.0	0.506
GIH	103	54.4	38.8	6.8	73.8	26.2	0.697
ITU	102	50.0	40.2	9.8	70.1	29.9	0.579
PJL	96	65.6	29.2	5.2	80.2	19.8	0.039*
STU	102	60.8	28.4	10.8	75.0	25.0	0.481

SLC29A3 C>T (rs10999776)

The homozygous wild-type CC was 52.2%, the heterozygous genotype was 37%, and the homozygous mutant TT was found to be 10.9%. Allele frequencies with C at 70.7% and for the minor allele T at 29.3% were different from only the AFR population. When compared with other populations, the frequencies were found to be similar (Table 4).

**Table 4:** Comparison of the genotype and allele frequencies of SLC29A3 rs10999776 C>T polymorphism in healthy population with 1000 genome populations *p < 0.05 was considered to be significant. AFR, African; AMR, American; BEB, Bengali in Bangladesh; EAS, East Asians; EUR, Europeans; GIH, Gujarati Indians in Houston, TX; ITU, Indian Telugu in the UK; N, number of subjects; PJL, Punjabi in Lahore; SAS, South Asians; SI, South Indian; STU, Sri Lankan Tamils in the UK

Present	N	Genotype frequency (%)			Allele frequency(%)		p-value
		CC	CT	TT	C	T	
SI	184	52.2	37.0	10.9	70.7	29.3	Ref
AFR	661	10.7	39.9	49.3	30.7	69.3	<0.0001
AMR	347	59.1	33.7	7.2	75.9	24.1	0.061
EAS	504	56.5	35.9	7.5	74.5	25.5	0.152
EUR	503	47.3	42.5	10.1	68.6	31.4	0.463
SAS							
BEB	86	55.8	36.0	8.1	73.8	26.2	0.444
GIH	103	52.4	41.7	5.8	73.3	26.7	0.499
ITU	102	52.9	38.2	8.8	72.1	27.9	0.722
PJL	96	43.8	44.8	11.5	66.1	33.9	0.273
STU	102	60.8	30.4	8.8	76.0	24.0	0.171

There were no gender-wise differences observed for the above-studied polymorphisms in healthy populations (Table 5).

**Table 5:** Gender-wise genotype and allele frequency distribution of gene polymorphisms in the healthy population *p < 0.05 was considered to be significant. SNP, single nucleotide polymorphism

SNP	Genotype frequency (%)			Allele frequency (%)		p-value
*DCK* rs80143932	CC	CG	GG	C	G	
Male (95)	88 (92.6)	6 (6.3)	1 (1.1)	182 (95.8)	8 (4.2)	-
Female (89)	84 (94.4)	4 (4.5)	1 (1.1)	172 (96.6)	6 (3.4)	0.673
*SLC29A1* rs760370	AA	AA	GG	A	G	
Male (95)	63 (66.3)	29 (30.5)	3 (3.2)	155 (81.6)	35 (18.4)	-
Female (89)	62 (69.7)	24 (27.0)	3 (3.4)	148 (83.1)	30 (16.9)	0.693
*SLC29A1 *rs9394992	CC	CT	TT	C	T	
Male (95)	52 (54.7)	35 (36.8)	8 (8.4)	139 (73.2)	51 (26.8)	-
Female (89)	43 (48.3)	41 (46.1)	5 (5.6)	127 (71.3)	51 (28.7)	0.698
*SLC29A1* rs10999776	CC	CT	TT	C	T	
Male (95)	52 (54.7)	32 (33.7)	11 (11.6)	136 (71.6)	54 (28.4)	-
Female (89)	52 (54.7)	36 (40.4)	9 (10.1)	136 (71.6)	54 (28.4)	1

The genotype and allele distributions in lung cancer patients are given in Table 6, and the present study did not find any gender-wise differences in frequencies for the studied SNPs in lung cancer patients (Table 7).

**Table 6:** Genotype and allele distribution of the studied gene polymorphism in lung cancer patients (N = 123) The genotype and allele frequencies were in Hardy-Weinberg equilibrium.

rs ID	Polymorphism	Genotype frequency (%)			Allele frequency (%)	
rs80143932	*DCK* C>G	CC 119(96.7)	CG 3 (2.4)	GG 1 (0.8)	C 241 (98.0)	G 5 (2.0)
rs760370	*SLC29A1* A>G	AA 93 (75.6)	AG 23 (18.7)	GG 7 (5.7)	A 209 (85.0)	G 37 (15.0)
rs9394992	*SLC29A1* C>T	CC 77 (62.6)	CT 35 (28.5)	TT 11 (8.9)	C 189 (76.8)	T 57 (23.2)
rs10999776	*SLC29A3* C>T	CC 72 (58.5)	CT 42 (34.1)	TT 9 (7.3)	C 186 (75.6)	T 60 (24.4)

**Table 7:** Gender-wise genotype and allele frequency distribution of gene polymorphisms in lung cancer patients p < 0.05 was considered to be significant. SNP, single nucleotide polymorphism

SNP	Genotype frequency (%)			Allele frequency (%)		p-value
*DCK* rs80143932	CC	CG	GG	C	G	
Male (78)	75 (96.2)	3 (3.8)	0 (0)	153 (98.1)	3 (1.9)	-
Female (45)	44 (97.8)	0 (0)	1 (2.2)	88 (97.8)	2 (2.2)	1.000
*SLC29A1* rs760370	AA	AA	GG	A	G	
Male (78)	60 (76.9)	12 (15.4)	6 (7.7)	132 (84.6)	24 (15.4)	-
Female (45)	33 (73.3)	11 (24.4)	1 (2.2)	77 (85.6)	13 (14.4)	0.842
*SLC29A1* rs9394992	CC	CT	TT	C	T	
Male (78)	52 (66.7)	22 (28.2)	4 (5.1)	126 (80.8)	30 (19.2)	-
Female (45)	25 (55.6)	13 (28.9)	7 (15.6)	63 (70)	27 (30)	0.053
*SLC29A1* rs10999776	CC	CT	TT	C	T	
Male (78)	47 (60.3)	27 (34.6)	4 (5.1)	121 (77.6)	35 (22.4)	-
Female (45)	25 (55.6)	15 (33.3)	5 (11.1)	65 (72.2)	25 (27.8)	0.347

Association of allele frequencies between lung cancer patients and healthy subjects

The SNP SLC29A1 rs9394992 showed a trend toward a protective effect against lung cancer after adjusting for age, gender, and smoking status (Table 8). There was no lung cancer risk observed with other SNPs (Table 9). The positive trend results did not reflect in the haplotype analysis for SLC29A1 rs9394992 C>T (Table 10).

**Table 8:** Association of SLC29A1 rs9394992 C>T polymorphism between lung cancer patients and healthy subjects *p < 0.05 was considered to be significant ^#^Adjusted for age, gender, and smoking status SNP, single nucleotide polymorphism

SNP	Lung cancer patients N (%) (N = 123)	Healthy subjects N (%) (N = 184)	p-value	Odds ratio (95% CI)	Adjusted odds ratio^#^ (95% CI)
*SLC29A1 *rs9394992 C>T					
CC	77 (62.6)	95 (51.6)	-	-	-
CT	35 (28.5)	76 (41.3)	0.036*	1.760 (1.06 to 2.90)	0.581^#^ (0.32 to 1.03)
TT	11 (8.9)	13 (7.1)	0.922	1.044 (0.44 to 2.46)	1.587 (0.58 to 4.29)

**Table 9:** Association of studied polymorphisms between lung cancer patients and healthy subjects *p < 0.05 was considered to be significant N (%), values in the brackets are presented as percentages SNP, single nucleotide polymorphisms

SNP	Lung cancer patients N (%) (N = 123)	Healthy subjects N (%) (N = 184)	p-value	Odds ratio	95% CI
*DCK* rs80143932 C>G					
CC	119 (96.7)	172 (93.5)	-	-	-
CG	3 (2.4)	10 (5.4)	0.255	0.4336	0.116 to 1.609
GG	1 (0.8)	2 (1.1)	1.000	0.7227	0.064 to 8.066
*SLC29A1* rs760370 A>G					
AA	93 (75.6)	125 (67.9)	-	-	-
AG	23 (18.7)	53 (28.8)	0.056	0.5833	0.333 to 1.019
GG	7 (5.7)	6 (3.3)	0.429	1.568	0.510 to 4.822
*SLC29A3* rs10999776 C>T					
CC	72 (58.5)	96 (52.2)	-	-	-
CT	42 (34.1)	68 (37.0)	0.438	0.8235	0.503 to 1.346
TT	9 (7.3)	20 (10.9)	0.232	0.6000	0.257 to 1.396

**Table 10:** Haplotypes association of SLC29A1 gene polymorphisms between lung cancer patients and healthy subjects p < 0.05 was considered to be significant.

S. no.	rs9394992 C>T	rs760370 A>G	Haplotypes	Total frequency	Frequency of		Chi square	p-value
					Lung cancer patients	Healthy subjects		
1	C	A	CA	61.4	65.1	58.9	2.413	0.1204
2	T	A	TA	22.0	19.8	23.4	1.117	0.2905
3	C	G	CG	12.7	11.7	13.4	0.376	0.5399
4	T	G	TG	3.9	3.3	4.3	0.348	0.5554

**Figure 1:**
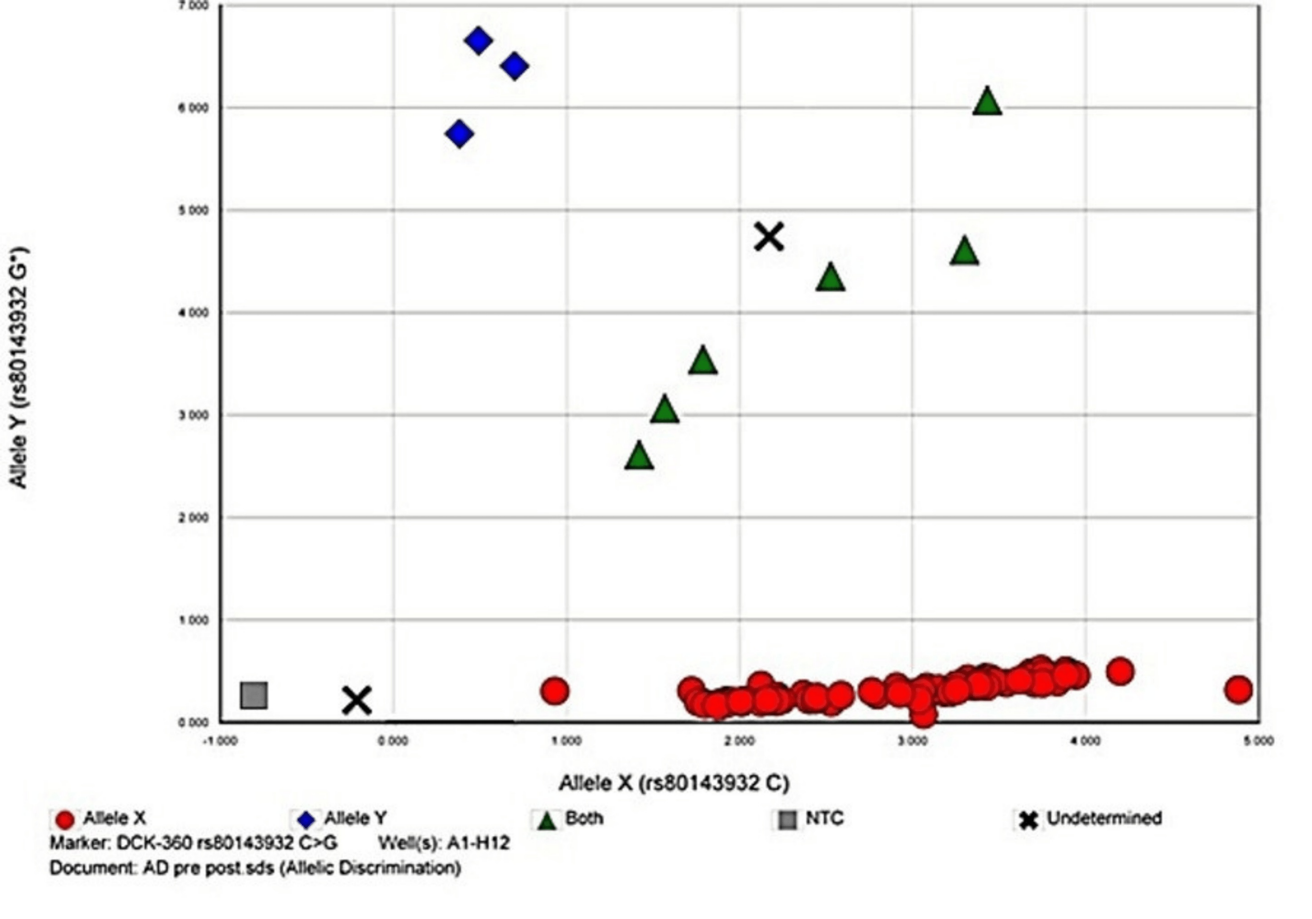
Allelic discrimination plot of DCK rs80143932 C>G polymorphism

**Figure 2:**
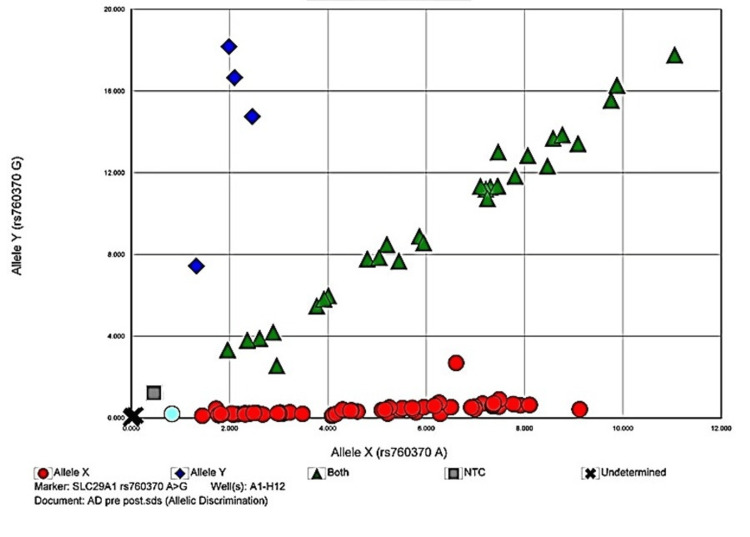
Allelic discrimination plot of SLC29A1 rs760370 A>G polymorphism

**Figure 3:**
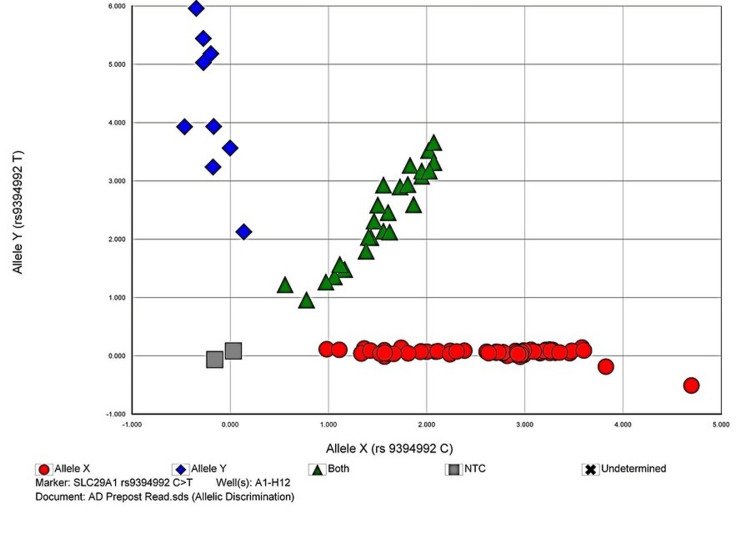
Allelic discrimination plot of SLC29A1 rs9394992 C>T polymorphism

**Figure 4:**
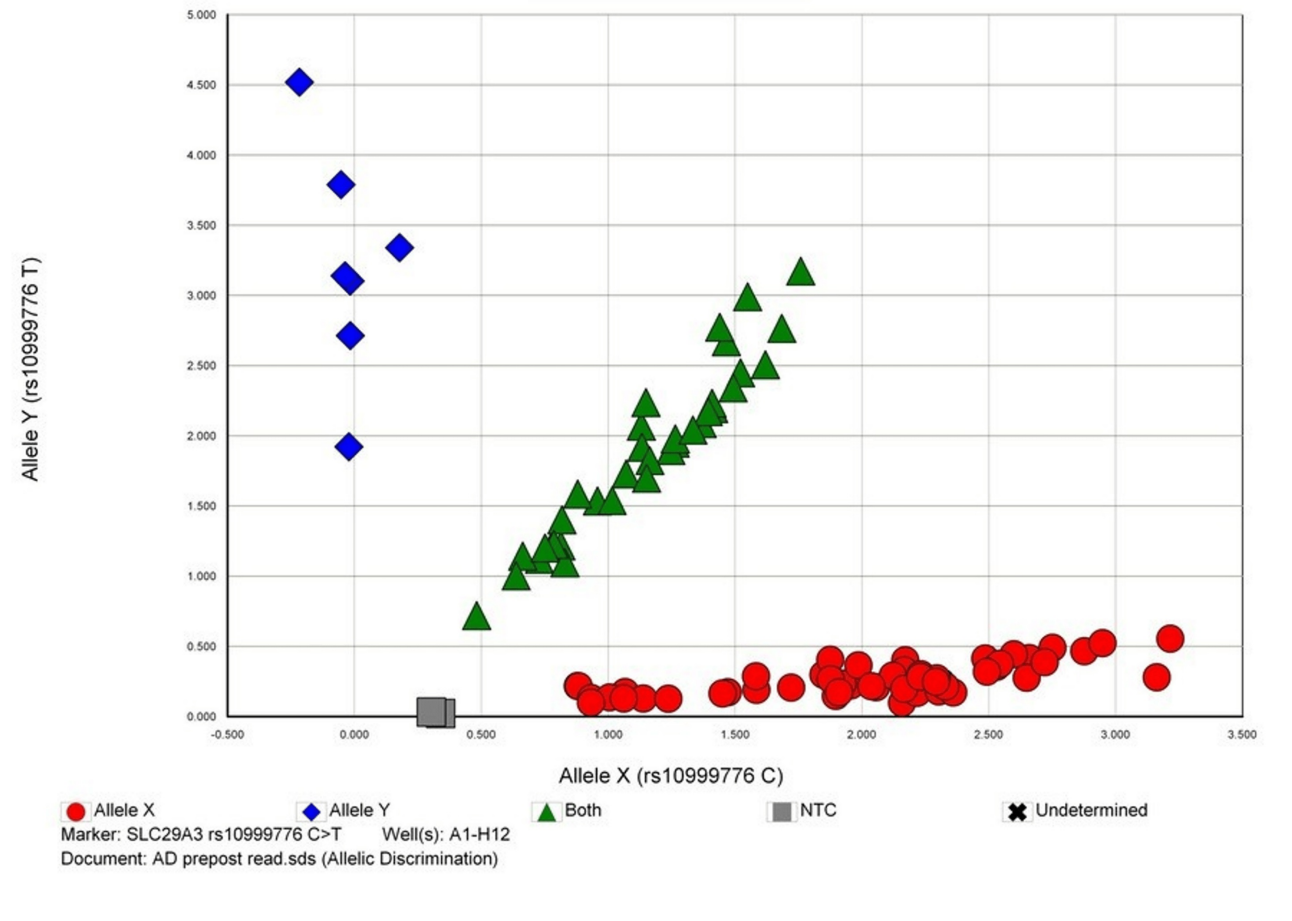
Allelic discrimination plot of SLC29A3 rs10999776 C>T polymorphism

The allelic discrimination plots to discriminate between two or more different alleles for the studied polymorphisms are given in Figures 1-4.

## Discussion

Non-small cell lung cancer is a prevailing and rapidly progressing challenge to modern medicine. Generalized medicine with conventional chemotherapy has comparatively shown poor clinical response rates and outcomes; the application of personalized medicine through individual genetic profiling for choosing the right treatment options has accelerated the successes in this area in terms of improved prognosis and patient outcomes.

The frequencies of polymorphic alleles vary considerably among global populations. These ethnic differences have a very crucial role in the benefitting treatment with anticancer drugs [23]. India is a place of diversity with a large number of population, including a mixture of various ethnic groups. Accordingly, variations at the genome level are expected between various groups, and South India stands to be the most diverse group in India [24]. The South Indian population in which the study was performed belonged to the Dravidian subgroup, and they constituted almost 25% of the total Indian population [25]. These diverse ethnicities might affect the outcomes and contribute to the differences in reported results [26]. Thus, ethnic differences with genetic markers can hence be used as curative in addition to diagnostic resources for achieving the true purpose of targeted, personalized chemotherapy. Ethnic differences were seen in drug response and adverse events [22].

DCK

The *DCK* enzyme is another important target molecule in gemcitabine metabolism wherein it causes activation of gemcitabine, and thus, any differences in this enzyme can lead to variations in drug response in patients on gemcitabine. The minor allele frequency (MAF) of *DCK* rs80143932 C>G was found to be higher when compared to AFR, EUR, and Indian Telugu in the UK (ITU) populations. On the other hand, it was lower in comparison with AMR, EAS, BEB, and Gujarat Indians in Houston, TX (GIH) populations. Interestingly, the MAF is fourfold higher in the EAS population (15.8%) than in our study (3.8%). Initially, this SNP was found by Shi et al. and team in the Asian population, wherein they reported that this SNP is in linkage with another SNP *DCK* -201 C>T. The variant allele of these linked promoter polymorphisms was higher as compared to this study and similar to the EAS population of 1000 genome data [4]. These linked variants were later studied in a mixed population with 90% of Caucasians. The reported MAF is less than the present study [5]. Similarly, the same authors had conducted another study in NSCLC patients and have found similar results [22]. This SNP has not been widely studied but is explored for its gene expression along with its linked SNP *DCK* -201 C>T. As they are very closely linked to each other, it is assumed that the presence of one SNP confirms the existence of the other SNP, which can also be applied to the effect that it produces in the clinical setting [4].

SLC

Transporter genes play a vital role in any drug to initiate its effect. Gemcitabine also comes under the category of being transported into the cell via transporters. Expression of the transporters can severely affect the cytotoxicity of gemcitabine. In the *SLC* family, the present study evaluated 3 SNPs, i.e., *SLC29A1 *rs760370 A>G, rs9394992 C>T, and *SLC29A3* rs10999776 C>T. The MAFs of the three SNPs are almost similar in this study. In addition, the variant allele of rs760370 A>G was similar compared to BEB, ITU, and STU (Sri Lankan Tamils in the UK). On the other hand, the frequencies were significantly different, as seen in AFR, AMR, EUR, EAS, and GIH populations. A Chinese study involving 115 healthy individuals has shown that the variant allele of rs760370 A>G is slightly higher as compared to the present study [27]. In a study including the Korean population, the frequencies of rs760370 A>G are higher than the frequencies of the present study [28]. Similarly, in a study that included healthy Chinese individuals, it was reported that the MAF was higher than the result from the present study [29].

*SLC29A1* rs9394992 C>T was also evaluated, and the genotype and allele frequencies were significantly different from the EAS and PJL populations. Interestingly, when compared with the above SNP, i.e., rs760370 A>G, the allele frequencies are similar in all the other populations from 1000 genome data with respect to this SNP. In the present study, an association of *SLC29A1* rs9394992 C>T between lung cancer patients and healthy subjects after adjusting for the odds ratio showed a trend toward a protective effect against lung cancer risk (Table 8, p = 0.036). The same was not reflected in the haplotype analysis. There is no available literature to substantiate these findings. The present results for lung cancer risk association need to be evaluated in future genomic studies to understand its relation with lung cancer.

For the SNP *SLC29A3* rs10999776 C>T, the allele frequencies were significantly different in the AFR population and were found to be similar when compared to the other 1000 genome populations, except that it showed a trend toward significant difference with the AMR population. Surprisingly, the African population had the C allele as the minor allele, which is the major allele in our study population as well as other HapMap populations, with a frequency of 30.7% and 69.3% for the C and T alleles, respectively [30]. The limitation of this study is the small sample size as to substantiate the results with the available scientific literature. Genomic-wide association studies would be more reliable in understanding the variations at the genetic level.

## Conclusions

The present study findings showed remarkable differences with respect to the genotype frequency of South Indians in healthy population as compared to other global populations. A trend toward protective effect was observed with respect to the SNP *SLC29A1* rs9394992 C>T in evaluating lung cancer risk association. Nevertheless, these results were not reflected in the haplotype analysis. Thus, there is a need to perform ethnic population-based genetic studies in larger samples to understand the role of genetic variability.
